# The impact of lenses on the seepage failure of tailings dam

**DOI:** 10.1371/journal.pone.0305425

**Published:** 2024-08-07

**Authors:** Hong Zhang, Quanming Li, Jiachen Wang, Botao Fu

**Affiliations:** 1 School of Energy and Mining, China University of Mining and Technology-Beijing, Beijing, China; 2 School of Civil Engineering, North China University of Technology, Beijing, China; 3 China Academy of Safety Science and Technology, Beijing, China; University of Science and Technology Beijing, CHINA

## Abstract

The presence of lenses such as tailings slurry, frozen soil, and saturated zones disrupts the continuity of tailings dams and their normal seepage patterns, elevating the seepage line of the dam body and significantly impacting local stability. This study, to investigate how lenses affect the stability and failure mechanisms of tailings dams, employs numerical simulation and physical models and constructs a model of the tailings dam, incorporating tailings clay lens and void lens, to investigate variations in hydraulic gradients, seepage velocities, seepage flow, pore water pressure, and the patterns of seepage failure. This research reveals that the tailings clay lens within the dam body increases the hydraulic gradient in its vicinity due to its low permeability and raises the phreatic line. As the tailings clay lens approaches the dam body, the phreatic line tends to escape along the upper part of the lens towards the dam surface. In addition, the void lens could lead to a more pronounced seepage gradient along its path on the dam surface, with a liquefaction beneath it. As the void lens nears the toe of the slope, the dam failure mode transitions from a step-like progressive failure to an arch-shaped settlement failure along the void lens.

## 1. Introduction

The local stability of tailings dams is crucial for their overall stability, with failures predominantly occurring in weak layers and their upper regions [1]. Influenced by factors such as mining methods, dam construction rate, climate, and routine management, the operation of tailing ponds inevitably leads to the formation of lenses such as slurry, frozen soil, ice layers, and voids within the tailings dam. This phenomenon has been confirmed in numerous geological exploration and research projects on existing tailings dams [2–5]. Compared in the surrounding tailings, the tailings dam lens is a weak area with spatial discontinuous and high water content and low mechanical strength. The presence of these lenses disrupts the continuity and normal seepage of the tailings dam, significantly impacting the stability of seepage and the local stability of the slope. One reason for the Brazilian tailings dam accidents is the prolonged water storage at high levels in weak areas, such as the lens within the tailings dam, leading to continuous seepage and erosion within the dam body, thereby resulting in large-scale collapses of saturated tailing materials when the dam ruptures [6, 7]. The collapse of the Karamken gold tailings dam in Russia is also related to changes in hydrological connectivity between the active layer at the bottom of the tailings pond and the frozen soil [8].

Characterized by its high concealment, prolonged development time, and tremendous destruction, seepage failure is one of the primary causes of accidents in tailings dams [9, 10]. Seepage failure in tailings dams can be classified into four types: piping, soil flow, soil flow on contact surface, and contact washing. However, single-layer seepage failure primarily manifests as the first two types mentioned above [11]. The key to understanding seepage failure in tailings dams lies in the formation of seepage channels, closely intertwined with the permeability, particle size, and thickness of tailings [12, 13]. Seepage induces significant settlement in tailings dams, with seepage flow carrying fine tailings towards lower areas, thus resulting in an increased content in fine tailings with depth and altering the particle size distribution of tailings [14].

OKEKE A C and Wang F [15] present a comprehensive experimental programme which evaluates the critical hydraulic and geometrical conditions for seepage-induced failure of landslide dams. WANG Y, et al. [16] were performed to investigate the seepage failure process in sandy gravels and fine-grained sands and the Reynolds number was obtained to describe the seepage regime. A coupled fluid-soil-structure simulator is developed combined the ISPH for fluid and the DEM for rubble mounds and caisson blocks by TSUJI K, et al. [17]. The models qualitatively reproduce the sand boiling and backward erosion in the opposite direction of the seepage flow. Liang Y, et al. [18] divide the erosion into 4 periods through the analysis of hydraulic head, flux and spring sand quantity: steady period before the superstratum breaking, period of the superstratum breaking, steady period after the superstratums breaking and period of the entire breaking. At present, research on the mechanisms of seepage failure in tailings dams primarily focuses on homogeneous single layers or the combined effects of multiple layers. However, there has been limited investigation into the influence of lenses on seepage failure in tailings dam. This paper establishes a numerical simulation model of the seepage field in tailings dams. It incorporates tailings clay lens and void lens to explore their impact on hydraulic gradients, seepage velocities, and seepage flow. Based on simulation results, working conditions significantly affecting seepage are simulated to construct physical models of seepage failure. By integrating the variation law of pore water pressure, this study examines the modes of seepage failure within tailings dams, aiming to unravel the effects of lenses on dam stability and analyze the mechanisms of failure under their influence.

## 2. Materials and methods

### 2.1 Experimental design

**Fig 1:**
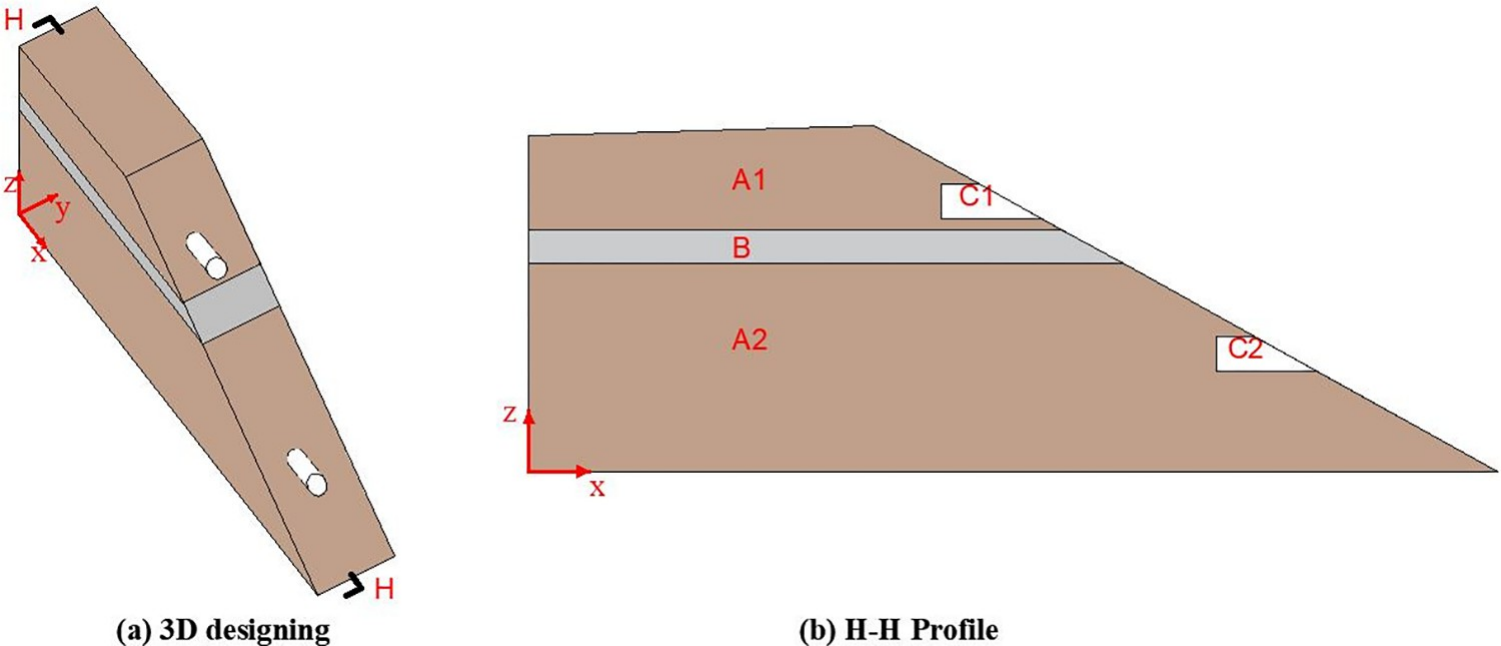
Numerical simulation model of the seepage field and the physical model of seepage failure in tailings dams. (A1, A2: tailings; B: tailings clay lens; C1, C2: void lens).

A conventional upstream tailings dam is used as a prototype to construct the model. The numerical simulation model of the seepage field and the physical model of seepage failure are illustrated in Fig 1, with the model dimensions detailed in Table 1. This study investigates the mechanism of seepage failure in tailings dams under the influence of tailings clay lens and void lens. In the model, the tailings clay lens is positioned at 60 cm, and voids are located at 28 cm and 74 cm. The study examines the development of hydraulic gradients, seepage velocities, seepage flow, and pore water pressure within the tailings dam when the water head height (H_w_) is set at 0.99 m (1 cm below the dam crest).

**Table 1:** Parameters of the numerical simulation model of the seepage field and the physical model of seepage failure in tailings dams.

Model Parameters		Dimensions
Dam height (H_d_) (m)		1.00
Dam length (L_d_) (m)		0.50
Crest width (Wd) (m)		0.10
Outer-slope ratio		1:1.8
Dry beach	Initial beach width (m)	1.00
	Slope (%)	3%
Lenses	Tailings clay lens height H_Le_ (m)	0.20
	Void lens radius R_Le_ (m)	0.05
	Void lens L_Le_ (m)	0.20
Water head height H_w_ (m)		0.99

In the numerical simulation model of the seepage field, the width of the tailings clay lens gradually increases using 4H_Le_ (where H_Le_ is the height of the lens) as a base. When approaching the dam slope, this base is appropriately reduced until reaching the slope. Based on the calculation results of the numerical simulation model, working conditions significantly affecting seepage in the tailings dam are selected to construct the physical model for seepage failure.

### 2.2 Design of numerical simulation model for seepage field

This paper employs the finite element method, widely utilized in both engineering practice and research of tailings dams, to analyze seepage patterns [19–22]. The constitutive model selected is the Duncan-Chang EB model, chosen for its ability to capture the nonlinearity, stress-dependent stiffness, and frictional characteristics of tailings [23–25]. The parameters are presented in Table 2. The Duncan-Chang EB model utilizes the tangent modulus E_t_ to represent the hyperbolic stress-strain relationship of the soil and the tangent bulk modulus B_t_ to depict the volume changes within the soil.


$$E_{t}=KP_{\alpha }{\left(\frac{\sigma_{3}}{P_{\alpha }}\right)}^{n}{\left[1-\frac{R_{f}(1-\sin\varphi )}{2c\cos\varphi +2\sigma_{3}\sin\varphi }\left(\sigma_{1}-\sigma_{3}\right)\right]}^{2}$$ (1)

Et——Tangent modulus, kN/m^2^;

σ_1_——Maximum compressive stress, kPa;

σ_3_——Minimum compressive stress, kPa;

P_a_——101.4 kPa, standard atmospheric pressure;

K——Modulus coefficient, dimensionless constant;

n——Modulus index, dimensionless constant where 0 < n < 1;

c——Cohesion, kPa;

ϕ ——Internal friction angle, °.

R_f_——Failure ratio; $R_{f}=\frac{{\left(\sigma_{1}-\sigma_{3}\right)}_{f}}{{\left(\sigma_{1}-\sigma_{3}\right)}_{u}}$ ${\left(\sigma_{1}-\sigma_{3}\right)}_{f}$——The value of $\sigma_{1}-\sigma_{3}$ when the specimen broken.

${\left(\sigma_{1}-\sigma_{3}\right)}_{u}$——the value of $\sigma_{1}-\sigma_{3}$ when axial pressure approaches infinity.


$$B_{t}=K_{b}P_{\alpha }{\left(\frac{\sigma_{3}}{P_{\alpha }}\right)}^{m}$$ (2)

B_t_——Tangent bulk modulus, kN/m^2^;

K_b_——Intercept of the straight line lg(B_t_/P_a_)~lg(σ_3_/P_a_);

m——Slope of the straight line lg(B_t_/P_a_)~lg(σ_3_/P_a_).

**Table 2:** Parameters for the numerical simulation model of seepage field.

Model Parameters		Tailings A1, A2	Tailings clay lens B
Elastic modulus (kN/m^2^)		11.02	6.32
Specific gravity		2.66	2.50
Cohesion (kPa)		5.5	36.6
Internal Friction Angle (°)		35	16.2
Duncan-Chang EB model	R_f_	0.70	0.67
	K	253	97
	n	0.79	0.67
	K_b_	95	24
	m	0.21	0.48
Horizontal seepage coefficient (m/d)		3.71	0.00016
Vertical seepage coefficient (m/d)		4.57	0.00035

### 2.3 Design of physical model for seepage failure

#### (1) Experimental materials

**Fig 2:**
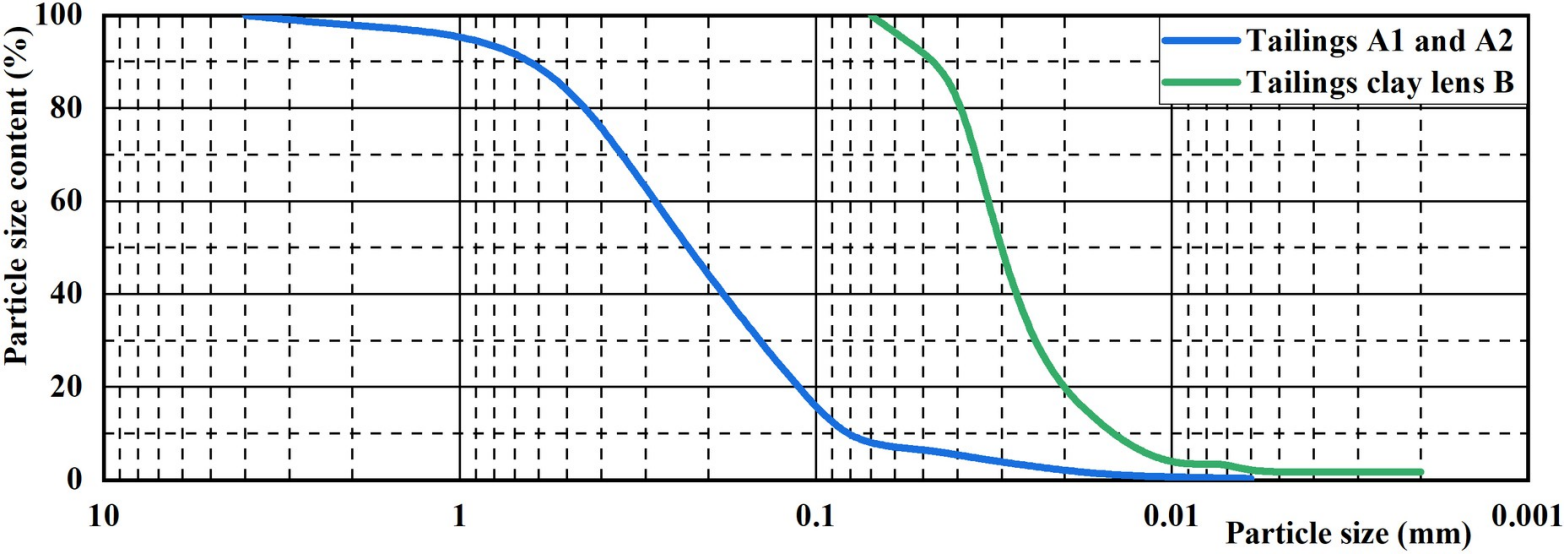
Particle size distribution of model tailings.

Iron ore tailings from a tailings dam are selected as the experimental material [26]. The distribution of particle size is depicted in Fig 2, and the key physical properties are outlined in Table 3. The tailings A1 and A2 at the dam predominantly comprise coarse particles, with a mean particle size (d_50_) of 0.21mm and an effective particle size (d_10_) of 0.09mm. Additionally, a small proportion of tailings clay is present in the tailings dam, with fines below 0.075mm constituting 7.57% of the total. Tailings clay lens B is composed of materials extracted from the tailings clay lens identified during the survey of the tailings dam. Its particle size is less than 0.075mm, of which the mean particle size (d_50_) is 0.031mm, and effective particle size (d_10_) is 0.015mm.

**Table 3:** Properties of the physical model for seepage failure in the tailings dam.

Material	Moisture Content (%)	Dry Density (kg/m^3^)	Natural Density (kg/m^3^)	Mean Particle Size d_50_ (mm)	Effective Particle Size d_10_ (mm)	Porosity Ratio (e)	Elastic modulus (kN/m2)	Cohesion (kPa)	Internal Friction Angle (°)
**Tailings A1 and A2**	15	1550	1690	0.21	0.09	0.81	11.02	5.5	35.00
**Tailings Clay Lens B**	15	1460	1580	0.031	0.015	0.76	6.32	36.6	16.20
**Void lens C1 and C2**	15	1165	1270	0.21	0.09	0.98	3.94	2.40	28.40

#### (2) Experimental equipment for seepage failure in tailings dams

**Fig 3:**
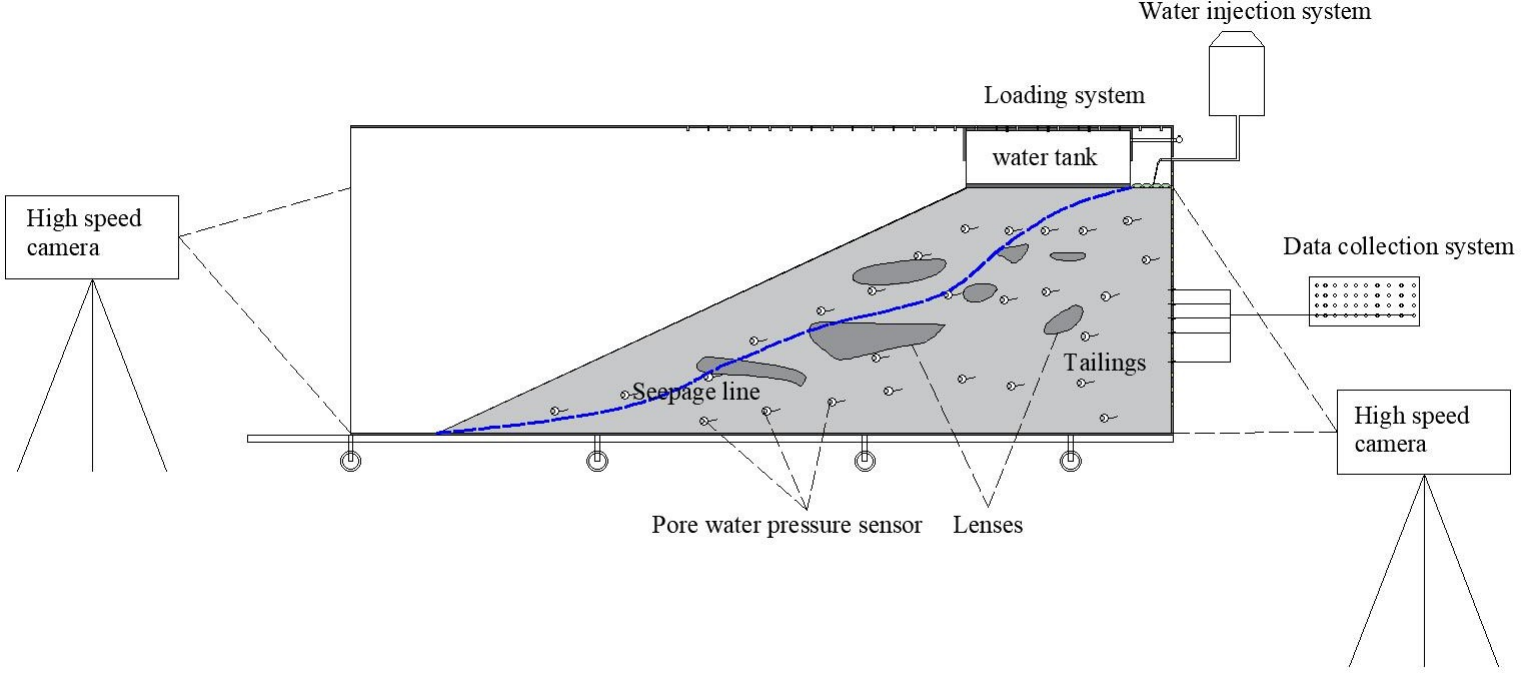
Chamber of physical model test for seepage failure.

Equipment suitable for conducting large-scale model experiments on tailings dams has been developed. This equipment comprises a loading system, water injection systems, slope failure observation, deformation testing, and other components, as depicted in Fig 3. The experimental setup can simulate the presence of special structures such as interlayers, weak underlying layers, and voids in tailings dams and investigate the mechanisms by which these factors affect the deformation and stability of the dams.

The equipment can apply pressure using pneumatic bags to meet the load requirements of different sub-dams during operation, enabling the study of the entire process of slope instability under the coupled fluid-solid interaction. The pneumatic bags are designed for easy removal. Once the pneumatic bags are removed, experiments on seepage failure in the natural state of the tailings dam can be conducted. The sidewalls of the physical model test chamber are reinforced with 4cm thick organic glass and cross braces to enhance their strength and prevent damage from excessive sidewall pressure.

#### (3) Construction of the physical model for seepage failure in tailings dams

**Fig 4:**
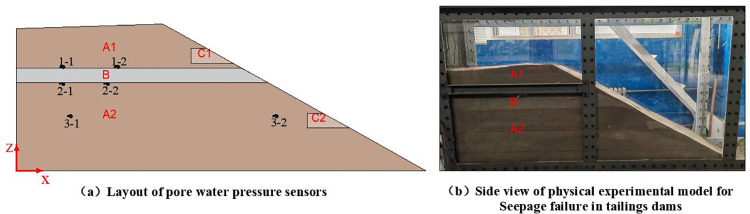
Physical model for seepage failure in tailings dams. (A1, A2 represent tailings; B represents tailings clay lens; C1, C2 represent void lens; 1–1~3–2 represent the positions of pore water pressure sensors).

The test model is constructed using a layered compaction method. In zone A2 of the model, coarse tailings are layered with a thickness of 20cm and stacked in three layers. At the 60cm mark, a 10cm-thick layer of tailings clay is placed as an interlayer (B). In zone A1, coarse tailings are stacked in layers of 20cm and 10cm, respectively. During construction, a void zone (C2) is established near the slope at the 28cm mark, with two pebbles placed for identification. A void lens (C1) is set up at the upper 8cm of the clay interlayer (B) in the same manner as C2. Additionally, pore water pressure sensors are installed at heights 40cm, 60cm, and 70cm, as shown in Fig 4(A).

The experiment adopts a method of gradually increasing the water head to simulate the seepage process in the tailings dam. Initially, the water level was raised to 0.975m, and the migration process of the water on the sidewall was observed, with the migration rate recorded. Subsequently, when the migration rate of water is below 0.5cm/min, the water head is raised in increments of 0.5cm until reaching 0.99m, at which point the model experiences seepage failure.

## 3. Analysis of numerical simulation results for seepage flow field in tailings dams

### 3.1 Analysis of seepage velocity

**Fig 5:**
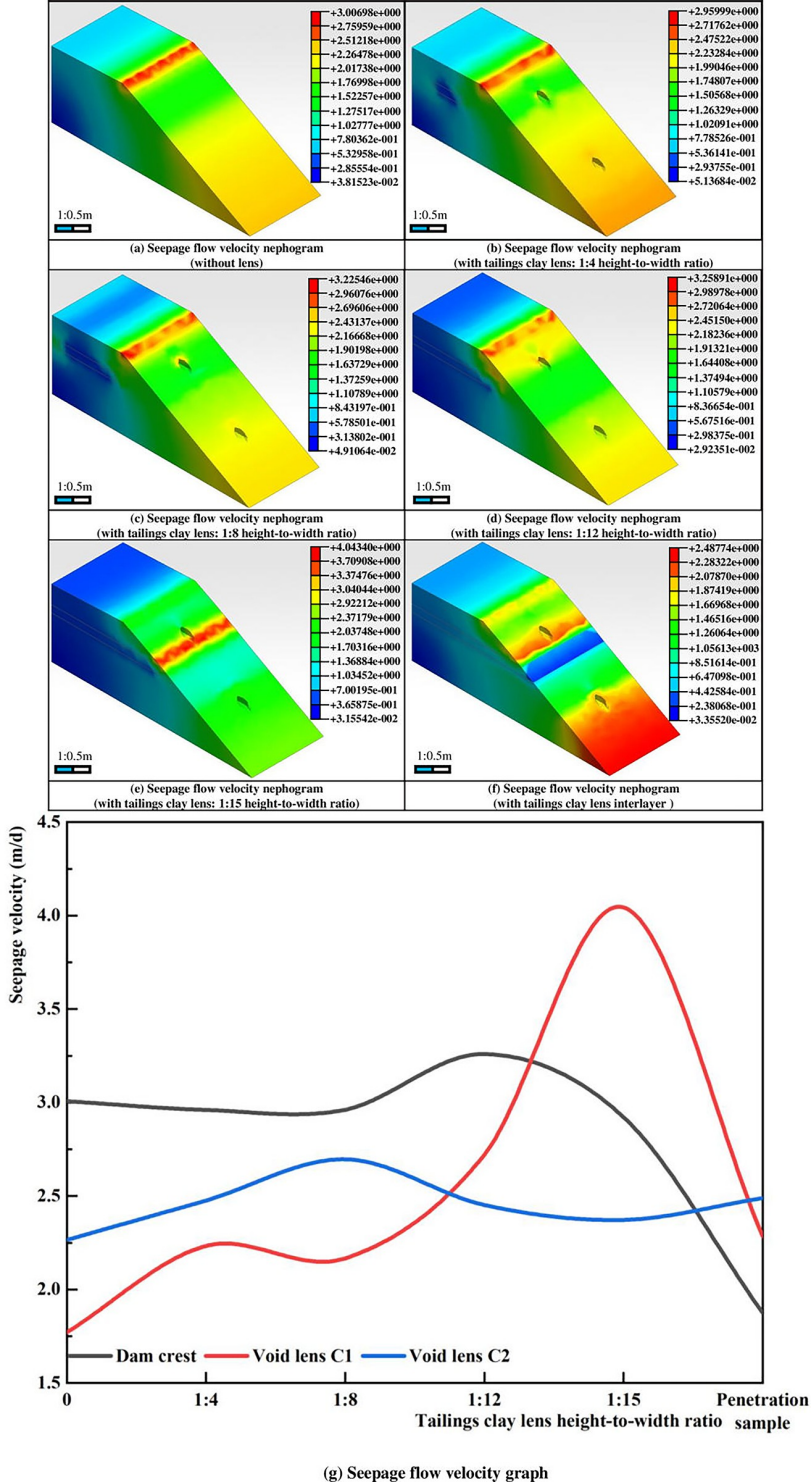
Seepage flow velocity nephogram and graph.

As depicted in Fig 5, the presence of lenses significantly impacts the seepage velocity in tailings dams. Without lenses, the seepage velocity of the dam body uniformly increases towards the dam slope, reaching a maximum velocity of 3.00698m/d at the dam crest, with a high-velocity zone of 2.01737~2.51218m/d formed from the bottom to 2/3 of the dam height.

As the size of the tailings clay lens within the dam body increases, the seepage velocity at the dam crest continuously rises. When the height-to-width ratio of the lens is less than 1:12, the maximum seepage velocity zone remains at the crest, and the presence of a tailings clay lens does not affect the seepage flow along the dam slope, as shown in Fig 5(G). However, when the height-to-width ratio of the lens reaches 1:12, the flow velocity at the crest reaches 3.25891m/d and flows downward along the slope, forming a "V"-shaped fluid interface at the top of the void lens C1; at this point, the center of the tailings clay lens is 0.52m away from the slope. As the size of the tailings clay lens continues to increase, the zone of maximum flow velocity in the dam body moves towards the lower part of the slope. When the height-to-width ratio is 1:15, the dam body forms a strip-shaped high seepage zone on the plane where the void zone C1 is located, with a seepage velocity of 4.0434m/d. When the tailings clay lens extends to the slope, the model forms a strip-shaped high-seepage zone at the plane where the void lens C1 is located and simultaneously forms a reverse "U"-shaped seepage interface at the top of the void lens C2; at this point, the maximum seepage velocity of the dam body is only 2.48774m/d.

Meanwhile, the presence of the tailings clay lens reduces the local seepage velocity within the dam body. Water circumvents the edges of the lens, creating an area of low flow velocity within it. When the height-to-width ratio of the lens exceeds 1:8, it begins to influence the overall seepage of the dam, forming a "ㄑ" shaped zone of reduced seepage within the dam body. As the height-to-width ratio of the lens continues to increase, the seepage velocity initially decreases before rising again. At a height-to-width ratio of 1:12, it reaches its minimum value of +2.92351e-002m/d, gradually increasing to +3.35520e-002m/d (when the lens extends to the dam slope).

This phenomenon can be attributed to several factors. Firstly, the tailings clay lens’s low permeability and high water retention capacity hinder the upward seepage of water, causing water to accumulate within the lens and form new seepage surfaces. This reduces the rate of downward seepage, leading to bypass flow and elevating the seepage line. As the tailings clay lens approaches the dam slope, the original seepage channels along the slope are disrupted, resulting in increased water pressure at the upper part of the lens and subsequent water seeping. Secondly, the presence of the void lens accelerates the seeping rate of water, creating a seepage gradient between the void zone and its surroundings and facilitating the flow of surrounding fluid toward the void zone. As the seepage surface of the slope rises, a significant seepage gradient forms at the location of the void lens.

### 3.2 Seepage flow analysis

**Fig 6:**
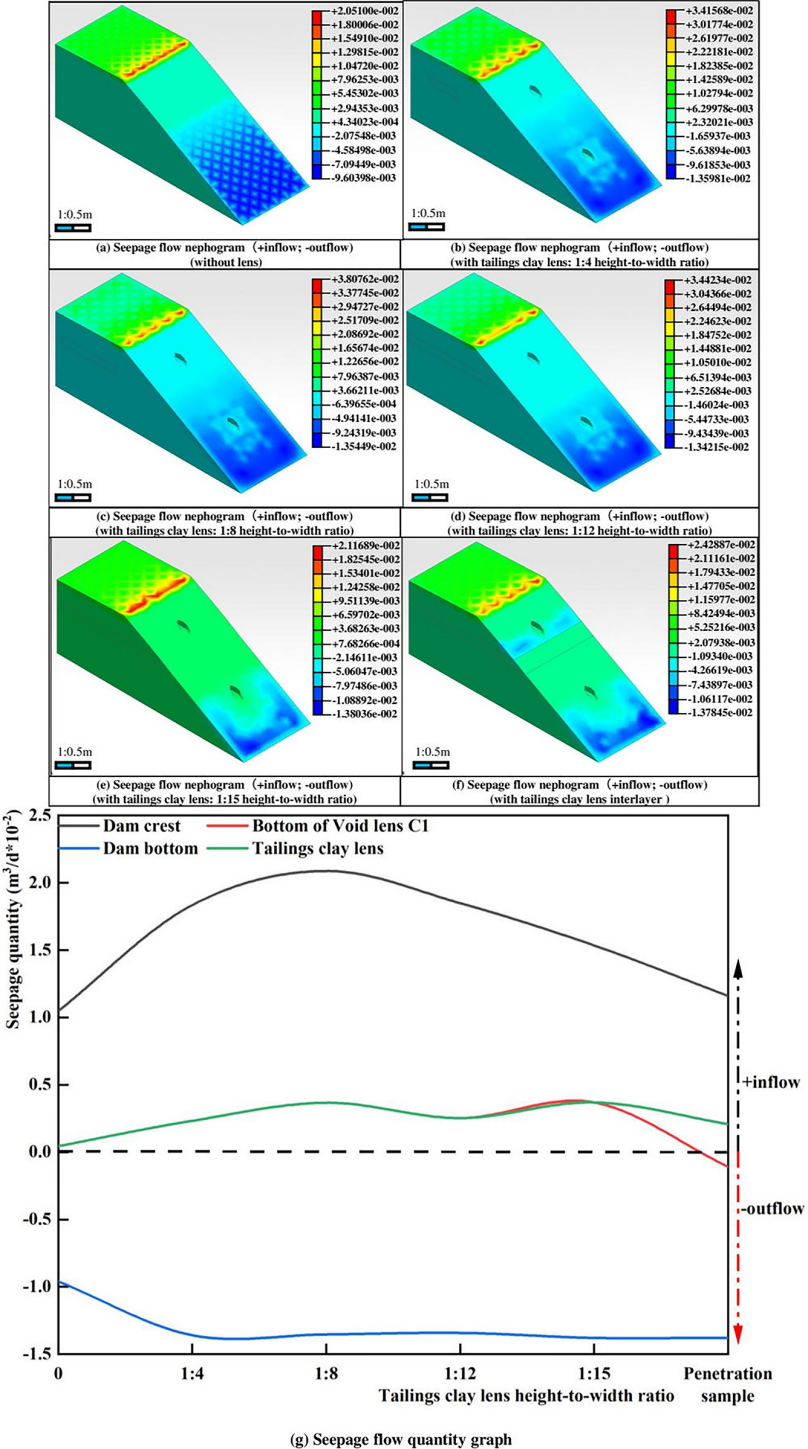
Seepage flow nephogram and graph (+inflow; -outflow).

As depicted in Fig 6, in the absence of any lens, water primarily seeps from the slope of the dam at a position located at two-thirds of the slope length from the bottom of the dam toe, with a maximum seepage flow rate of 9.60398e-003m^3^/d.

When the height-to-width ratio of the tailings clay lens is less than 1:12, the seeping position along the dam slope is identical to that of the model without a lens. However, the seepage flow is concentrated around the edges of the void lens C2 and at the base of the dam, with a peak rate of seepage flow at 1.35981e-002m^3^/d. Upon reaching a height-to-width ratio of 1:12 for the tailings clay lens, its continued extension toward the slope gradually shifts the seeping position along the slope closer to the dam base. Consequently, the seepage flow steadily increases, eventually forming a "U" shaped seepage surface. When the tailings clay lens extends to the outer slope of the dam, water forms a seepage surface on both sides of the void lens on the dam slope. At this stage, the rate of seepage flow is 1.37845e-002m^3^/d, slightly lower than the maximum rate of 1.38036e-002m^3^/d at a **height-to-width** ratio of 1:15.

In summary, the presence of the lens significantly affects the seeping location and seepage flow along the dam slope. The seepage flow at the dam base increases with the size of the tailings clay lens. However, when the tailings clay lens approaches the dam slope, the seepage flow at the dam base decreases, while the seepage flow at the upper part of the tailings clay lens increases, as shown in Fig 6(G). The void lens alters the seeping range along the dam slope, changing it from two-thirds of the way down from the dam’s base to a "U" shaped seeping at the dam base, with the maximum seepage flow occurring below the void zone.

### 3.3 Hydraulic gradient analysis

**Fig 7:**
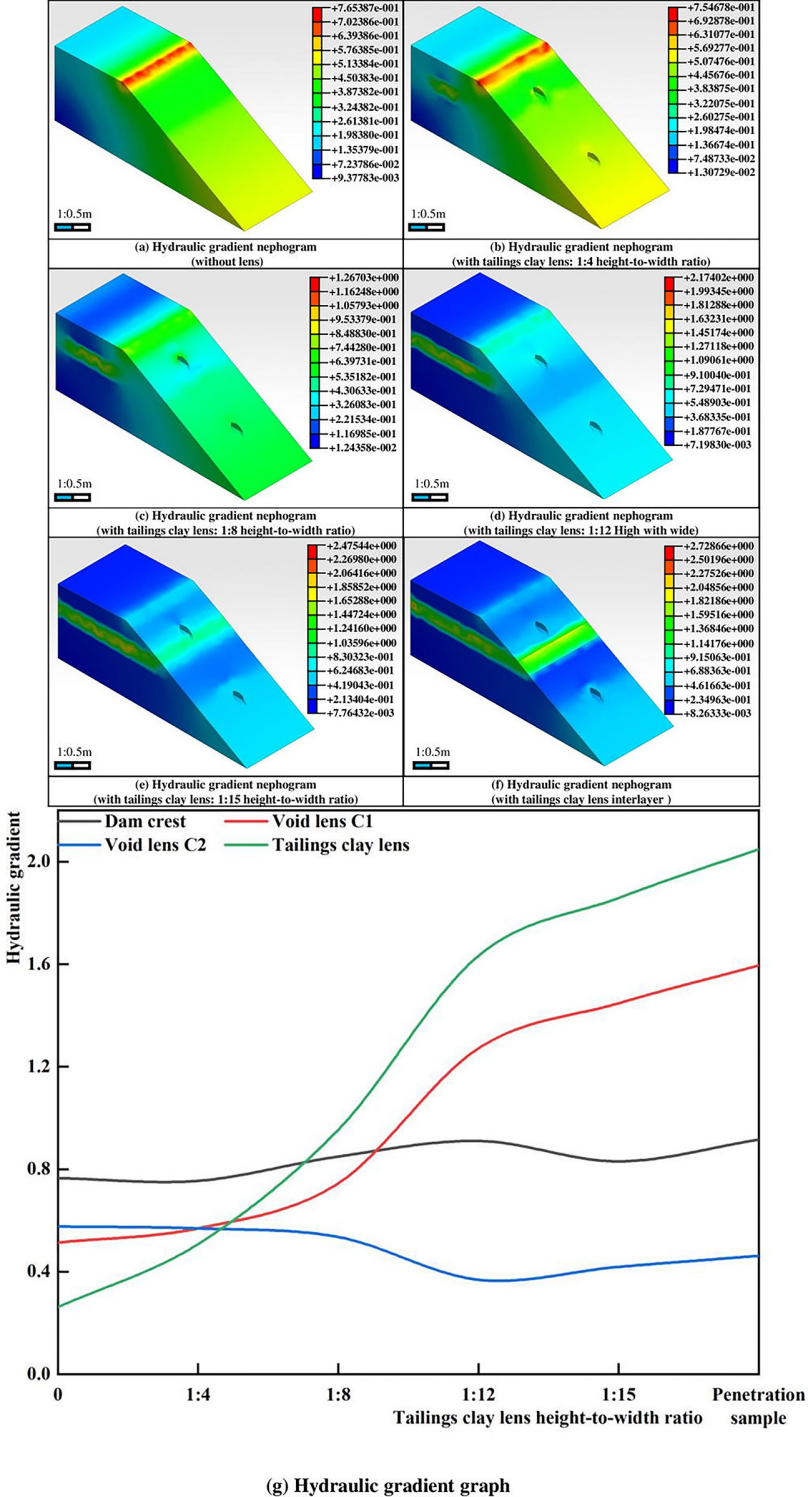
Hydraulic gradient nephogram and graph.

With a water level of 0.99m, the hydraulic gradient of the dam body without any lenses is shown in Fig 7(A). In the absence of lenses, the highest hydraulic gradient in the dam body is at the dam crest, reaching a maximum value of 0.766. The gradient values along the slope are lower than those at the base of the slope. When lenses are present, the hydraulic gradient of the dam body increases with the size of the tailings clay lens. The presence of the tailings clay lens leads to an increase in hydraulic gradient around its area while simultaneously reducing gradients in its vicinity. As the size of the tailings clay lens continues to increase, the hydraulic gradient within the lens also rises, while gradient values in the surrounding areas decrease and expand outward, as shown in Fig 7(G). When the tailings clay lens extends to the outer slope of the dam, the low-gradient area spreads to two-thirds of the dam body, reaching a minimum value of 8.26333e-3.

When the height-to-width ratio of the tailings clay lens is less than 1:12, a high-gradient area appears within 1/3 of the arc-shaped area above the void lens. When the height-to-width ratio of the tailings clay lens is greater than 1:12, an arch-shaped high-gradient area forms on the slope above the void lens and gradually spreads downward. Meanwhile, when the tailings clay lens approaches the outer slope of the dam, a low-gradient area forms in the upper part of the void lens, extending to the interval between the tailings clay lens.

The reason behind this lies in the water-retentive nature of the tailings clay lens, which increases its own hydraulic gradient value, thereby reducing the gradient within the area that is 0.2H_d_ (H_d_ is the model height) lower. When the tailings clay lens approaches the dam body, its low permeability increases the upper water pressure. However, the pressure difference formed by the void lens results in the formation of an arch-shaped high-gradient area on the slope with the void zone as the center.

## 4. Analysis of seepage failure in tailings dam with a physical model

### 4.1 Patterns of seepage failure

**Fig 8:**
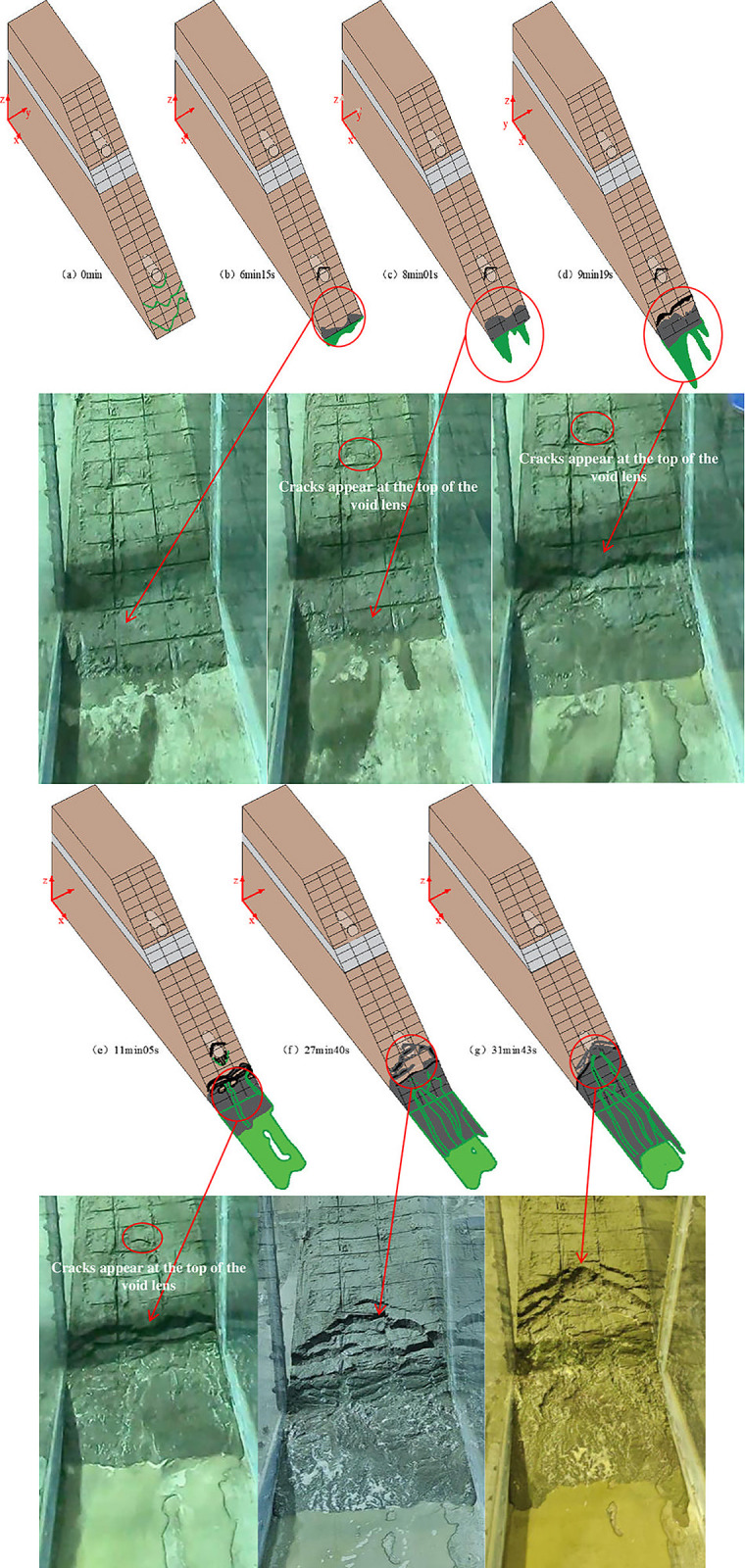
Complete process of seepage failure in tailings dam.

The moment when water starts to flow out is considered the initiation of seepage failure, and the failure process was recorded using a GoPro camera, as shown in Fig 8. Through an analysis of the seepage failure in the tailings dam, the process is divided into three stages:

Phase 1 (initiation of seepage failure, Fig 8(A) and 8(B)): The dam surface starts to exhibit marsh-like characteristics, accompanied by turbid water seepage emerging from the dam base. A notable "marshification" phenomenon occurs beneath the void lens C2, forming a rippled and liquefied surface. At 6 minutes and 15 seconds, turbid water begins to flow out from the right side of the dam toe, which has a width of approximately 16.7 cm. With continuous outflow, the tailings on the right side of the dam toe undergo reverse erosion, resulting in turbid tailings flow under the influence of water and gravity. Simultaneously, arc-shaped cracks develop in the upper part of zone C2 of the dam body, indicating the formation of an initial failure surface. It is moderate in comparison with the development rule of seepage velocity and hydraulic gradient at the dam bottom in Figs 5(G) and 7(G). As shown in Figs 5(G) and 7(G), On the slope below the tailings clay lens, an arch-shaped high seepage velocity and high-gradient area appear on the top of the void lens C2, which further indicates that the seepage surface of the dam slope will appear in this area.

Phase 2 (development of seepage failure, Fig 8(C) and 8(F)): The dam body undergoes stepped-like seepage failure, gradually transitioning into arc-shaped failure. Firstly, as the water flow at the dam base increases, the width of the seep surface at the dam toe expands to 36 cm, forming a U-shaped erosion surface. This observation aligns with the seepage nephogram depicted in Fig 6(F). Secondly, a fracture collapse occurs at the 25 cm mark on the dam surface, causing the tailings within the 20 cm of the dam base to collapse outward. As the tailings at the bottom move, the cracks at the top of C2 enlarge. Under the combined influence of water flow and gravity, the collapsed lower tailings form a tailings flow, spreading outward. The failure of the dam gradually forms a stepped-like morphology and develops upstream. Thirdly, the stepped-like collapse on the dam surface within a range of 25 cm below the arc-shaped crack changes to an arc-shaped collapse. Tailings flow continues to spread outward from 10 to 30 cm on the dam surface, while tailings collapse downward under the influence of gravity from 30 to 50 cm. Water flows out along the vertical marking lines beneath C2, forming noticeable gullies above the tailings flow. Remarkably, at 11 minutes and 5 seconds, clear signs of water erosion appear about 16 cm from the right side of the dam surface, creating gullies, which indicates the presence of seepage channels above.

Phase 3 (stable stage of seepage failure, Fig 8(G)): The arc-shaped failure progresses to the upper part of C2, stabilizing the failure mode. At 31 minutes and 43 seconds, the dam exhibits arch-shaped collapses along the top of C2, while the dam corresponding to the upper part of C2 remains stable. Two flow channels extend from C2 to the left and right sides, forming gullies downward on the dam surface Then, the model stabilizes once again.

Based on the starting point and development position of the gullies in the model, it is inferred that seepage channels have formed at C2, allowing seepage water to escape onto the dam surface and form gullies within the tailings flow. Through the entire process of seepage failure, it is speculated that seepage failure mainly occurs in the upper part of the void lens. Additionally, during the initial stage of seepage failure, a rippled and liquefied surface is observed in the area of C1, which is consistent with the nephogram depicted in Fig 6(F) for the downward seepage flow in the C1 area.

To further determine the location of the seepage channel, after allowing the model to stand still for 1 hour, the tailings at C2 of the dam body are cleared. Care must be taken during the clearing process so as not to disturb the upper tailings in the void zone. Two pebbles in C2 are then placed approximately 10 cm below its bottom. Upon clearing the tailings at the void zone, it is observed that water is seeping from the junction of the void lens and the dam body, while no water seeping is observed in the tailings on either side of the void zone.

**Fig 9:**
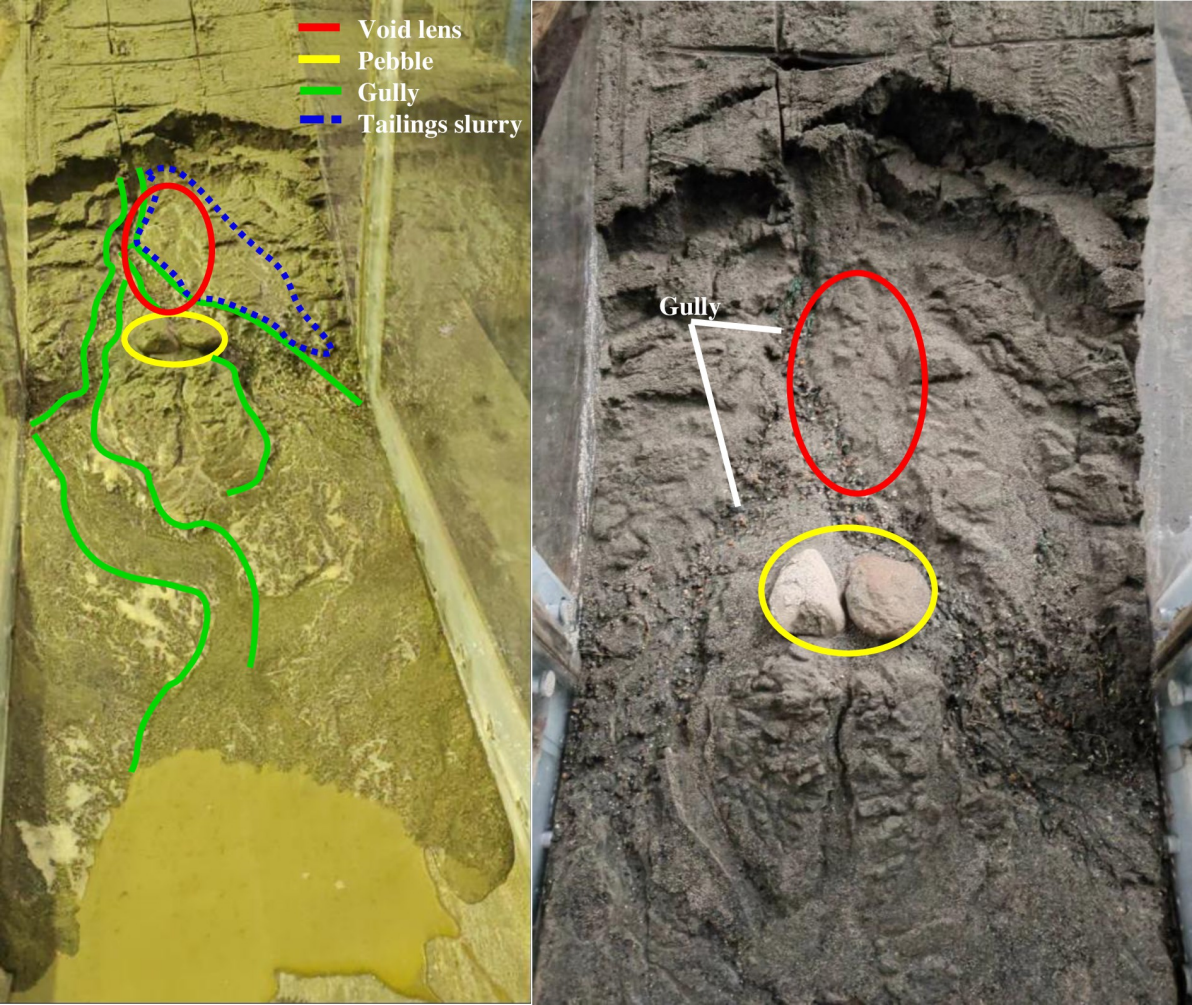
Final form of the second seepage failure.

After another 1-hour rest, the tailings dam was brought back to the water level at the time of the seepage failure, and the seepage locations on the dam surface were observed. As shown in Fig 9(A), water seeped downward from above C2, forming gullies at the original location of the void zone. The tailings on both sides of the void zone continued to collapse downward, but no water seeped from within the dam body. This observation, in conjunction with the seepage failure process of the tailings dam depicted in Fig 8, confirms that the seepage channel exit of the tailings dam is located in the void zone lens.

### 4.2 Development pattern of pore water pressure

**Fig 10:**
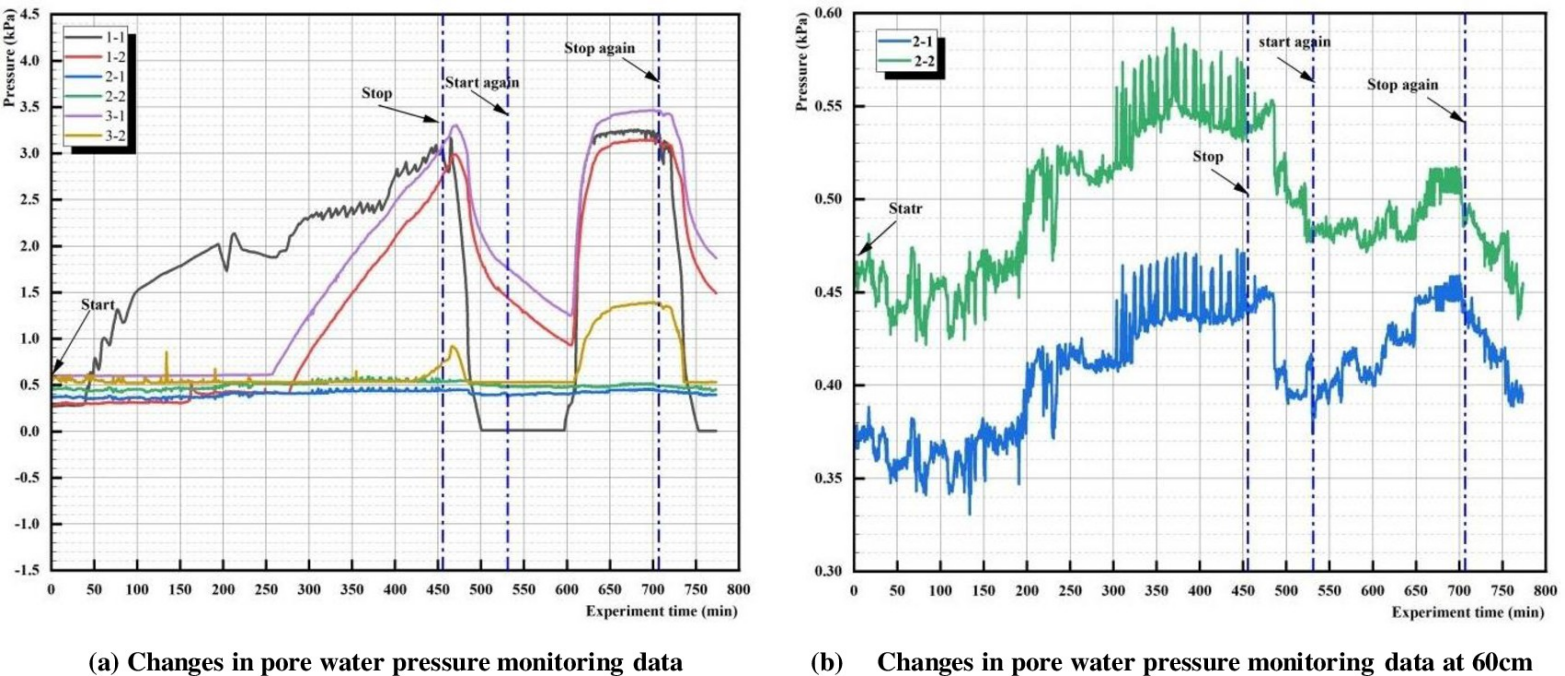
Pore water pressure monitoring data.

Analysis of pore water pressure monitoring data at depths of 70cm, 60cm, and 40cm (from bottom to top) was conducted, as shown in Fig 10.

During the first experiment, sensor 1–1 exhibited pressure changes 39.5 minutes after the experiment commenced, indicating water had migrated to that location. With increasing seepage, the pressure at this location continued to rise. At 182.5 minutes, it migrated to the bottom of the tailings clay lens interlayer B at sensor 1–2, with a migration time of 143 minutes, indicating significant water retention in the tailings clay lens interlayer. Influenced by the interlayer, the downward seepage decreased, and it reached sensor 3–1 at 60cm at 256.5 minutes. Despite the water head remaining unchanged, the sensor pressure continued to rise as water seeped. At 466 minutes, the pore water pressure sharply decreased, indicating rapid water loss at the sensor. Coupled with the water seeping from the slope at C2 at 455 minutes, it indicated that after water seeped from the dam surface, the remaining water in the dam increased its seeping, ultimately leading to seepage failure of the dam surface at 466 minutes.

During the second experiment, it took about 30 minutes from the onset of the pore water pressure change to reach the peak pressure, which was 14.67 times shorter than the 440 minutes in the first experiment. This indicated that after the formation of the first seepage channel, the water in the reservoir quickly drained along the formed seepage channel. After reaching peak pore water pressure, due to the constant water head, the internal water pressure of the dam reached stable seepage; thus, the pore water pressure remained constant. After stopping the experiment, the water pressure at the dam crest disappeared, and the internal water of the dam quickly drained along the seepage channel, causing rapid dissipation of pore water pressure.

Comparing the curves of the two stops in 8(a), after both experiments were stopped, the pore water pressure decreased after 15 minutes, and the dissipation trend of pore water pressure after the peak point was consistent.

**Fig 11:**
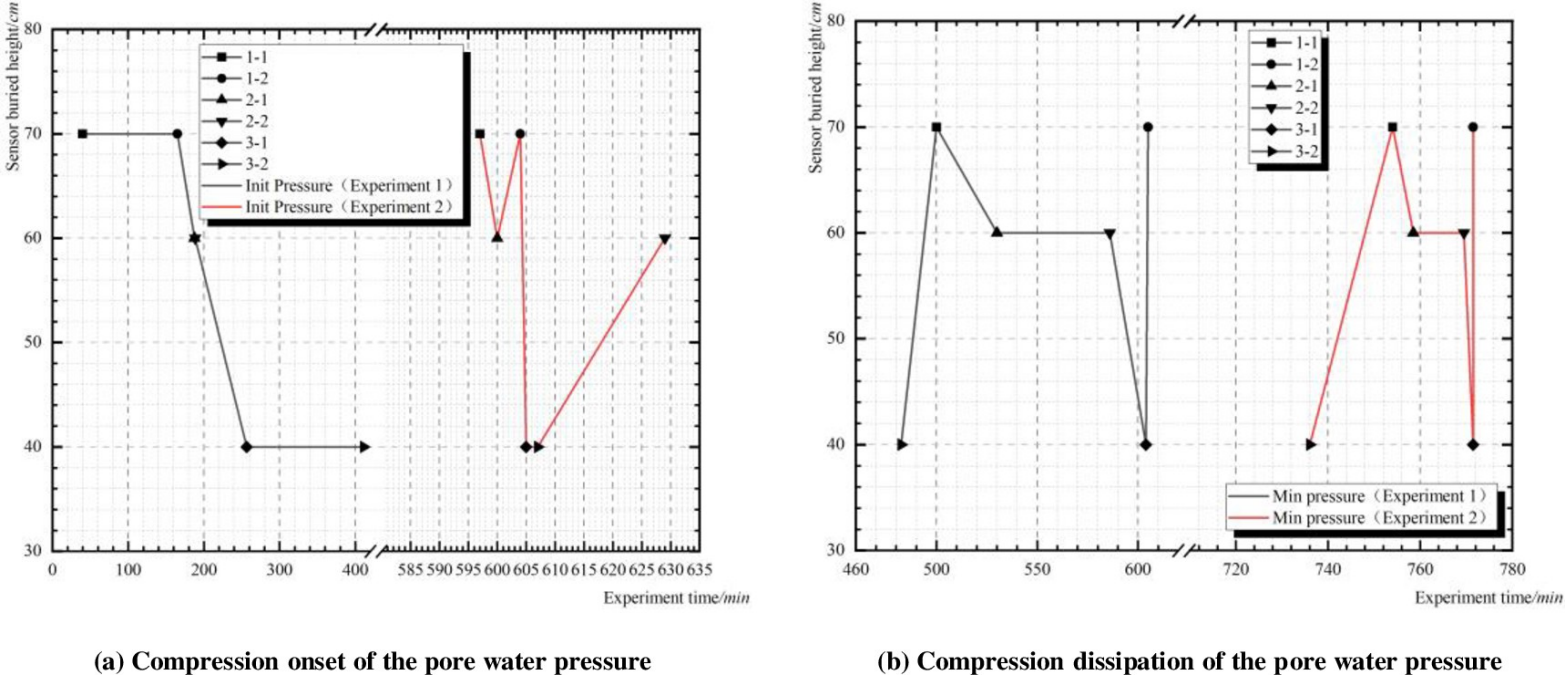
Pore water pressure variation over time.

A comparison of the starting point and dissipation time of pore water pressure changes during the two experiments is shown in Fig 11.

As depicted in Fig 11(A), during the first experiment phase, the sequence of onset times for changes in pore water pressure was 1–1–1–2–2–1–2–2–3–1–3–2. Initially, as there was no seepage channel formed within the dam, the water encountered the clay interlayer B, causing a decreased rate in the vertical seepage. Consequently, the pore water pressure change at 1–2 occurred earlier than at 2–1. Additionally, influenced by the water-retaining properties of interlayer B, a new seepage surface formed within interlayer B, covering the range of pore water pressure sensors 2–1 and 2–2, resulting in similar onset times for pressure changes at 2–1 and 2–2. This migration pattern is also corroborated in Fig 5.

During the second experiment phase, the sequence of onset times for changes in pore water pressure data was 1–1–2–1–1–2–3–1–3–2–2–2. In this scenario, the rate of vertical seepage within the dam was higher than the horizontal rate, diminishing the water-retaining effect of the tailings clay lens B. Since a seepage channel had been established within the dam during the first phase of the experiment, for the second experiment, water within the dam preferentially seeped along this channel. Based on the changing pattern of sensor data, a seepage channel likely formed within the dam between 1–1 and 2–1, leading to earlier pressure changes at 2–1 compared to 1–2. Subsequently, water seeped sequentially from 2–1 to 3–1 and 3–2. Therefore, the seepage channel within the dam may be inferred as follows: 1–1–2–1–3–1–3–2.

As depicted in Fig 11(B), an analysis was conducted on the sequence of pore water pressure reduction following two seepage failures. After the seepage damage, water within the dam escaped through the seepage channels, resulting in a gradual reduction in pore water pressure within the dam. Following the first seepage failure, the pore water pressure sequentially decreased as follows: from the point of seepage termination to the point of seepage initiation, then vertically, and finally horizontally (3–2–1–1–2–1–2–2–3–1–1–2). The reason for this sequence lies in the fact that the point of seepage termination is located near the void zone of C2. Once seepage occurs, water rapidly seeps from the dam surface, resulting in an immediate decrease in pore water pressure at that location. This leads to the termination point decreasing in pressure earlier than the initiation point. Based on the sequence of seepage and the positions of pore water pressure sensors, it can be inferred that the seepage channels formed within the dam could be 1–1–2–1–3–1–3–2 and 2–2–3–1–3–2.

After the second seepage failure, the reduction sequence of pore water pressure remained consistent with the first experiment. This indicates that the dam body continued to evacuate water along the seepage channels formed during the first phase. Overall, from the combined processes of the two experiments, it can be inferred that when seepage failure occurs in the tailings dam, seepage points will emerge in the weak areas of the da m slope [27].

## 5. Conclusion

This paper adopts a combined approach of experimental and numerical simulation methods to establish a tailings dam model incorporating the tailings clay lens and void lens. The study investigates the impact of the lens on the hydraulic gradient, seepage velocity, seepage flow, pore water pressure variations, and the seepage failure mode of the tailings dam. The findings are summarized as follows:

1. The presence of the lens alters the normal seepage mode within the dam. While the tailings clay lens elevates the seepage line of the dam, it also creates new areas of high hydraulic gradient, influencing local seepage pressures. The void lens induces significant seepage gradients within the dam, leading to a liquefied surface beneath the void lens and its lower part.
2. The lens plays a significant role in shaping positon of seepage channels within the tailings dam. They can alter the direction of these channels. Additionally, the void lens creates weak areas within the dam, where if the hydraulic pressure exerted by the tailings dam is sufficient to breach and carry tailings out of the slope, seepage points will emerge in those areas.
3. The presence of the void lens changes the seepage failure mode of the tailings dam. When the void lens is located near the slope toe, the initiation point of seepage failure occurs at the top of the void lens [1, 28–30]. This results in a transition from a step-like failure to an arch-shaped settlement failure along the position of the voids.
